# Correlation Between DNase I Hypersensitive Site Distribution and Gene Expression in HeLa S3 Cells

**DOI:** 10.1371/journal.pone.0042414

**Published:** 2012-08-10

**Authors:** Ya-Mei Wang, Ping Zhou, Li-Yong Wang, Zhen-Hua Li, Yao-Nan Zhang, Yu-Xiang Zhang

**Affiliations:** 1 Department of Biochemistry and Molecular Biology, Cancer Institute, Capital Medical University, Beijing, China; 2 Department of Bioinformatics and Computer Science, School of Biomedical Engineering, Capital Medical University, Beijing, China; 3 Microarray Core Facility, Capital Medical University, Beijing, China; Peking University Health Science Center, China

## Abstract

Mapping DNase I hypersensitive sites (DHSs) within nuclear chromatin is a traditional and powerful method of identifying genetic regulatory elements. DHSs have been mapped by capturing the ends of long DNase I-cut fragments (>100,000 bp), or 100–1200 bp DNase I-double cleavage fragments (also called double-hit fragments). But next generation sequencing requires a DNA library containing DNA fragments of 100–500 bp. Therefore, we used short DNA fragments released by DNase I digestion to generate DNA libraries for next generation sequencing. The short segments are 100–300 bp and can be directly cloned and used for high-throughput sequencing. We identified 83,897 DHSs in 2,343,479 tags across the human genome. Our results indicate that the DHSs identified by this DHS assay are consistent with those identified by longer fragments in previous studies. We also found: (1) the distribution of DHSs in promoter and other gene regions of similarly expressed genes differs among different chromosomes; (2) silenced genes had a more open chromatin structure than previously thought; (3) DHSs in 3′untranslated regions (3′UTRs) are negatively correlated with level of gene expression.

## Introduction

In the era of functional genomics, the challenge is to elucidate gene function, regulatory networks and signaling pathways [1]. Since regulation of gene expression *in vivo* mainly occurs at the transcriptional level, identifying the location of genetic regulatory elements is a key to understanding the machinery regulating gene transcription. A major goal of current genome research is to identify the locations of all gene regulatory elements, including promoters, enhancers, silencers, insulators and boundary elements, and to analyze their relationship to the current annotation of human genes [2], [3]. In recent years, many genome-wide strategies have been developed for identifying functional elements. However, no method yet has the resolution to precisely identify all regulatory elements or can be readily applied to the entire human genome. The classical method of mapping DNase I hypersensitive sites (DHSs) by Southern blotting has been used to identify many different types of genetic regulatory elements [4], but it can only be applied to one small region of the genome at a time. Chromatin immunoprecipitation with microarray detection (ChIP-chip) can define the global locations of regulatory factors [5], [6], [7], but is more suitable for studying known factors, and requires high quality ChIP antibodies. More recently, new methods have been described that work by capturing a library of chromatin with DNase I-digested ends, and by using massively parallel signature sequencing (MPSS) for sequencing (DNase-seq), or labeling and hybridization to tiled microarrays (DNase-chip) [8], [9]. Crawford et al. produced approximately 230,000 sequence tags and identified an estimated 20% of sites in their DNase-seq experiments [10], while their DNase-chip strategy covered 1% of the genome [11]. Boyle et al mapped open chromatin using a DNA library from single DNase I cleavage ends and next-generation sequencing (NGS) [12], while Sabo et al generated a DNase I library of DNA fragments (<∼1200 bp) released by two-cleavage ‘hits’ occurring next to each other and identified DNase I hypersensitive sites (DHSs) using microarrays [13], [14].

The introduction of next generation sequencing (NGS) technology is one of the major breakthroughs in recent genomics research [15], [16], [17], [18]. Generally a DNA library of short fragments (100–500 bp) is required for NGS. Thus, methods capable of generating large numbers of short DNA fragments are advantageous for NGS. We speculated that DNase I double-hit fragments of 100–300 bp would resist mechanical shear better than longer sequences during DNase I digestion, and this would help us lower background noise. In addition, the short DNA fragments would be easily purified, and could be used for NGS library preparation, thus greatly simplifying library preparation and sequencing.

In the present study, we enriched short DNA fragments (100–300 bp) released by DNase I digestion and generated a DNA library from human HeLaS3 cells. For convenience we call this method the “Short DHS assay”. We identified 83,897 DHSs in 10,505,607 DHS tag sequences with high sensitivity and specificity. By combining whole-genome data from the Short DHS Assay and expression microarrays, we detected a specific correlation between DHS location and gene expression. Our data suggest that the Short DHS Assay is straightforward and should be a valuable tool for preparing DNA libraries for global identification of gene regulatory elements.

## Materials and Methods

### Cell Culture and Synchronization

HeLa S3 cells were purchased from the Cell Culture Center of Peking Union Medical College. They were cultured in F-12 Nutrient Mixture (Ham) (Invitrogen, USA), containing 10% fetal bovine serum (FBS), penicillin/streptomycin at 37°C and 5% CO_2_ and used in experiments at a density of 5×10^6^ cells/ml. To remove the background introduced by actively dividing cells, we used the standard approach for synchronizing cells in G1 by serum deprivation. Cells were arrested in F-12 Nutrient Mixture with 0.2% FBS (24 h) [19], [20], and then placed on ice prior to harvesting nuclei.

### Nuclear extraction and DNase I digestion

Cells were spun down, washed with ice-cold PBS (2,000 rpm for 3 minutes at 4°C), and the pellets were resuspended in 500 µl of 1×Lysis RSB buffer [21] [250 mM sucrose, 10 mM Tris pH 7.4, 10 mM NaCl, 3 mM MgCl_2_, 0.1 mM PMSF] and gently lysed with 0.2% Nonidet P-40 in RSB buffer, by incubating on ice for 10 min. We sedimented nuclei at 2,000 rpm for 10 min at 4°C, and washed the pellets with 500 µl RSB buffer. We then sedimented them again at 2,000 rpm for 10 min at 4°C, and resuspended them gently in 500 µl of ice-cold 1×reaction buffer (50 µl 10×DNase I buffer (Roche Molecular Biochemicals), 450 µl water), using pipette tips with cut off ends, and spun again at 2,000 rpm for 10 min at 4°C. They were digested with RNase-free DNase I (Roche Molecular Biochemicals) (10 U/ml) for 10 min at 37°C in 400 µl volumes of 1×DNase I buffer (40 µl 10×DNaseI buffer (Roche Molecular Biochemicals), 360 µl water). DNase I digestion was stopped with 5% SDS, 50 mM EDTA (pH 8.0). Proteinase K (25 µg/mL final concentration) was added and the mixtures were incubated overnight at 55°C. 4 µl RNase A (10 mg/ml, Ambion) was added to each sample and the samples were further incubated at 37°C for 30 min and DNA extracted using the standard phenol-chloroform technique. Care was taken to use cut-off tips and very gentle pipetting to reduce non-specific DNA sheering. After precipitation the DNA was resuspended in 50 µl of ddH_2_O.

### DNase I digestion of control DNA

We isolated nuclei twice as described above, and purified DNA by phenol-chloroform extraction and ethanol precipitation, followed by dissolving the DNA overnight in 50 µl of ddH_2_O. We retained one DNA preparation as the untreated control, and then digested the other with DNase I (10 U/ml) at 37°C for 10 min in 400 µl volumes of DNase I 1×buffer to generate pools of random control fragments. The digestion was stopped with 50 mM EDTA (pH 8.0) and the DNA recovered by ethanol precipitation.

### Isolation of specific and nonspecific DNase I fragments

We isolated specific and nonspecific DNA fragments by Gel purification. After gel-electrophoresis, the target DNA bands were cut from agarose gels and purified with a QIA Quick Gel Extraction Kit (Qiagen). We purified DNA fragments of 100–300 bp and also fragments larger than 300 bp as a control.

### DNA Library preparation and high-throughput sequencing

**Figure 1:**
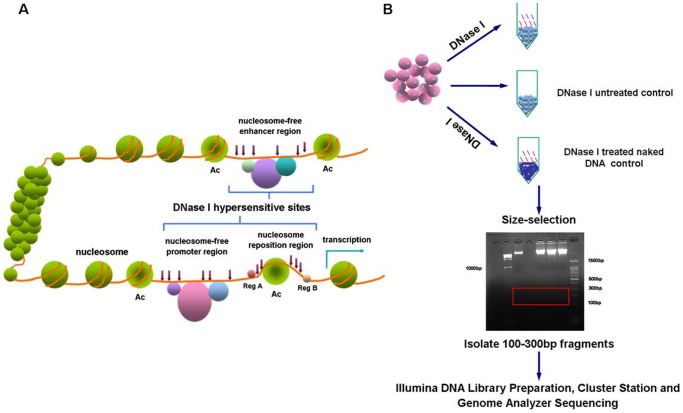
High-resolution mapping of accessible chromatin in human cells using the Short DHS Assay. (A) DNase I hypersensitive sites (DHSs) within chromatin. (B) Isolation of specific and nonspecific DNase I fragments. Short fragments (100–300 bp) released by DNase I treatment were isolated by size selection and gel purification; DNA fragments of the same size were also purified from DNase I-untreated control and DNase I-treated naked DNA. Gel-purified DNA fragments were end-repaired followed by cluster generation and massive parallel sequencing using an Illumina Genome Analyzer GA II.

The gel-purified DNA products were modified for Illumina Whole-Genome Chromatin IP sequencing using an Illumina Genomic DNA Sample Prep kit as follows: the size-selected DNAs were end-repaired by T4 DNA polymerase and phosphorylated by T4 DNA polymerase and T4 polynucleotide kinase. The products were incubated with Klenow DNA Polymerase (Illumina) to generate 3′ adenine overhangs and then ligated to Illumina adapters, which contain 5′ thymine overhangs. The adapter-ligated products were purified on QIAquick spin columns (Qiagen), PCR-amplified with Phusion DNA Polymerase (Finnzymes) for 10 cycles using Illumina's genomic DNA primer set. The PCR products were purified on QIAquick and MinElute columns (Qiagen).The quality of the DNA was assessed and quantified using an Agilent DNA 1000 Series II assay and NanoDrop ND-1000 spectrophotometer (Thermo Scientific) and the DNA was diluted to 10 nM. Cluster generation and sequencing were performed using a Standard Cluster Generation kit and a Cycle Solexa Sequencing kit on the Illumina Cluster Station and Illumina Genome Analyzer I following the manufacturer's instructions [22]. A diagram of the Short DHS Assay is presented in Figure 1 (Figure 1A, 1B). Sequencing was done by the Research & Cooperation Division, BGI-Shenzhen.

### Real-time PCR for validation of DHSs

Real-time PCR was used to verify the reliability of the Short DHS assay for identification of DHSs. We randomly selected six captured DHSs, and designed PCR primers to match each DHS upstream and downstream sequence (Table S1). Each primer pair was designed to generate a 100–200 bp product by using Primer3 [23]. DNase I-treated and non-digested DNA was quantified in triplicate. The DNase-treated DNA, non-digested DNA and DNase-treated naked DNA control were each fractionated into 100–300 bp and >300b mixtures as above. Samples (10 nanograms each) were stamped onto 96 plates, and primer/Brilliant II Fast SYBR® Green QPCR Master Mix (Stratagene, Agilent Technologies) was added. All PCR reactions were performed on an Mx3000P PCR machine (Stratagene). Pilot PCRs performed in triplicate generated highly reproducible results (SD<0.2). Only dissociation curves with single peaks, indicating specific amplification, were used in the analysis. ΔCt values were determined by subtracting the Ct value of each DNase I concentration from the non-DNase I-treated control Ct value for each primer set.

The PCR mixtures contained: template DNA 1 µl (10 ng/µl); 2×SYBR Green mix 12.5 µl; 10 µM forward primer 1 µl; 10 µM reverse primer 1 µl; reference dye 0.375 µl; H_2_O 9.125 µl added to a total volume of 25 µl. The thermal cycling parameters were as follows: initial denaturation at 94°C for 3 min; 35 cycles of denaturation at 94°C for 30 sec, annealing at 64°C for 30 sec, and extension at 72°C for 30 sec; and a final extension at 72°C for 10 min.

### Whole-genome gene expression analysis

Total RNA was extracted from HeLa S3 cells with a To TALLY RNA™ Total RNA Isolation Kit (Ambion). Then 500 ng of HeLa S3 total RNA was amplified and labeled using an Illumina® TotalPrep™ RNA Amplification Kit (Ambion). 1.5 µg samples of purified and labeled cRNAs were directly hybridized to a Human HT-12 v3 Expression Bead Chip (Illumina). After sample hybridization, washing, blocking, and staining with Streptavidin-Cy3 (GE Healthcare Bio-Sciences Corp.), the chip was scanned with a Bead Array Reader (BeadStation500, Illumina). The HT wash buffer, block and Hyb buffer were supplied with the Illumina Gene Expression buffer kit. Data outputs were analyzed with the Illumina BeadStudio software.

### Primary data analysis

Primary sequencing data analysis consisted of: (i) Basic information analysis; (ii) Peak region scanning, including peak region detection, peak counts, average peak length, median peak length; (iii) The associated genes with sample peaks; (iv) Depth of coverage distribution of the samples' mapped reads in the gene region; (v) GO function notability enrichment analysis of peak-related genes. We also compared our data to two independent sets of HeLa S3 DHS data downloaded from the UCSC website (DNase I Hypersensitivity by Digital DNaseI from ENCODE/University of Washington (http://genome.ucsc.edu/cgi-bin/hgTrackUi?db=hg19&g=wgEncodeUwDnase)), in order to confirm the reliability of our method. Digital DNase I digestion of these two DHS data sets was performed by DNase I digestion of intact nuclei and isolating DNaseI ‘double-hit’ fragments as described in Sabo et al. [13].

### Comparison of genome annotation and gene expression data

We analyzed the distribution of DHSs in cis-elements and functional regions such as promoters, CpG islands, downstream 20 k regions, GC boxes, and regions from transcription start sites (TSS) to transcription end sites (TESs, also called transcription termination sites, TSSs). We then explored the relationship between the distribution of DHSs and levels of gene expression. Expression values from 5 to 11 are raw log2 ratio-transformed data from gene expression arrays [12]. Gene expression was classified as silenced (<5), low (5–6), medium (6–10), high (10–11) and very high (>11) according to the log2 expression value.

## Results

### Genome-wide distribution of DHSs

#### Identification of DHSs in human HeLa S3 cells by high-throughput sequencing

Among 14,284,385 sequence tags generated by high-throughput sequencing, we identified 10,505,670 unique mapped reads (35 bases in length) in the human genome (Table S2). The genome-wide distribution of the sequence reads is shown in Table S3. The proportion of reads in exon regions is 8.81%, and the enrichment factor is 6.3. The proportion of reads in intron regions is 40.59%, and the enrichment factor is 1.2.

**Figure 2:**
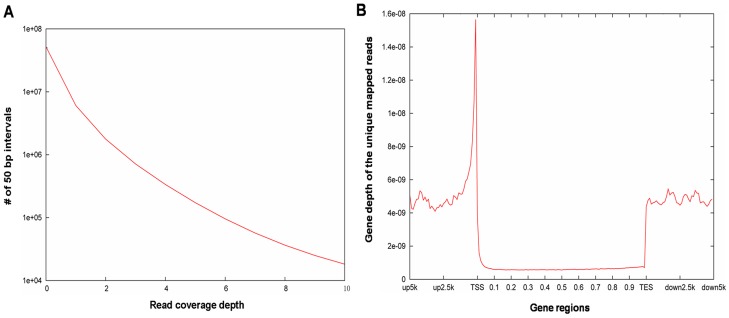
Genome-wide depth of coverage of unique mapped reads. (A) Depth of coverage of unique mapped reads. Samples randomly selected from the same number of reads (which can be compared to the reference genome. [refGene.txt.g from http://hgdownload.cse.ucsc.edu/ goldenPath/hg18/database/refGene.txt.gz].The genome-wide read depth of coverage was then calculated by counting the number of reads in 50 bp intervals. (B) Gene depth of the unique mapped reads. Regions from 5 K upstream of genes and 5 K downstream of transcription start sites (TSS) were divided into 40 equal parts; the gene itself was also divided into 100 equal parts, and the reads were then mapped to each region. The degree of coverage was calculated from the following formula: total tag number of a region/(total number of sample tags×length of region in bp). Horizontal axis TSS = transcription start site, TES = transcription termination site.

After filtering, and aligning read tags to reference sequences http://hgdownload.cse.ucsc.edu/goldenPath/hg18/database/refGene.txt.gz), we calculated the average read coverage for all non-overlapping 50-bp slide windows of the genome. Sequence read depth of coverage is shown in Figure 2A. We also examined the depth of coverage of unique mapped reads in the genes. We found that unique mapped reads exhibited marked aggregation around TSSs (Figure 2B). It has been reported that the TSSs of essentially all highly expressed protein-coding genes, and possibly all expressed genes, are marked by DHSs [12]. The results in Figure 2B imply that DHSs are also specially enriched in regions proximal to TESs, and depleted in distal intergenic regions. We also observed an increased density of DHSs immediately 3′ of gene TESs. The regions immediately downstream of TESs may be involved either in transcription termination, or in antisense transcription [24], [25].

#### Genome-wide distribution of DHSs in regulatory sequences and functional regions associated with genes

We compared the coverage of single sample mapped reads in different functional areas of the genome. We found that 11.76% of HeLa S3 DHSs were situated within the proximal promoter regions 4 K upstream of known genes, 7.29% in the 4 K regions downstream of known genes, 8.81% in exons, 40.59% in introns, and 31.55% in intergenic regions (Figure S1A).

Unique mapped reads in repeats are shown in Figure S1B. We found that 29.57% of these reads mapped to SINE/Alu, and 17.02% to LINE/L1. The L1 and Alu families harbor the most common mammalian long and short interspersed elements [26]. Alu sequences contain several functional transcription factor binding sites and are present in the 5 kb upstream regions of the TSSs of about 14,000 genes [27]. Our distribution suggests that the genome-wide DHS DNA library generated by the Short DHS assay contains many DHSs related to Alu elements.

### Validation of DHSs by real-time PCR

**Figure 3:**
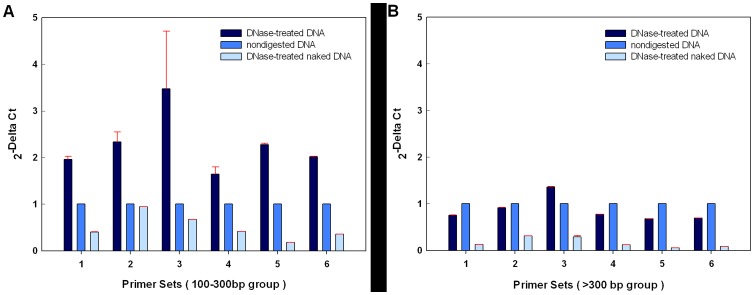
Validation of DHSs by Real-time PCR. DHS sensitivity was determined by comparing signals generated by Real-time PCR using ten nanograms of DNase-treated DNA, non-digested DNA or DNase-treated naked DNA as templates. DNA fragments from each group were divided into sub-fractions of 100–300 bp and >300 bp, respectively, and used as templates for PCR. Six primer sets were used for Real-time PCR, and amplicons of about 100∼200 bp were expected from each set. (A) Real-time PCR quantification of 100–300 bp DNA fragments released by DNase I cleavage. (B) Real-time PCR quantification of DNA fragments longer than 300 bp. The 2 −ΔΔCt method was used to calculate the differences among the different groups. The results are averages of three independent experiments. Data are means ± SDs.

We used real-time PCR to confirm the DHSs identified by the Short-DHS assay. We randomly selected six captured DHSs from the DNase-treated DNA, non-digested DNA and DNase-treated naked DNA control. Each group was fractionated into a sub-group of 100–300 bp and one of >300 bp. Figure 3A, B represents the mean ± SD enrichment of three independent biological replicates. The 2 ^−ΔΔCt^ method [28], [29] of monitoring the digestion of DNA fragments by DNase I allows straightforward comparison of the cycle threshold values of the DNase I-treated and of the untreated fragments of genomic DNA. The 100–300 bp DNA fragments released from DNase I-treated nuclei were more highly enriched than the 100–300 bp fragments of the non-digested DNA, and DNase I-treated naked DNA controls (Figure 3A). In the >300 bp groups, the tags in the DNase I-treated naked DNA group were much less numerous than in the groups from DNase I treated or untreated nuclei (Figure 3B), confirming the efficacy of DNase I treatment. Taken together, the results indicate that the 100–300 bp DNA fragments released from DNase I-treated nuclei are enriched in DHSs, and that the Short DHS assay is a sensitive and specific method of identifying DHSs.

### Location of DHS peaks within the annotated genome

#### Use of peak-calling algorithms to identify potential peaks referred to as enriched regions

Using software MACS 1.4.0, we identified 83,897 statistically significant peaks of reads (p-value<1e-04), occupying 1.35% of the human genome (Table S4). Average peak length was 482 bp, and median peak length 619 bp (Figure S2A). The read numbers of the peaks and the peak numbers were added using cumulative statistics (Figure S2B). This showed that each peak had more than 6 reads. The peak distribution over the whole genome was then used to analyze the distribution of gene-associated DHSs.

#### Overall analysis of peak-related genes

We used two independent HeLa S3 DHS datasets downloaded from the UCSC website (DNaseI Hypersensitivity by Digital DNase I from ENCODE/University of Washington (http://genome.ucsc.edu/cgi-bin/hgTrackUi?db=hg19&g=wgEncodeUwDnase)) as positive controls and named them Control 1 and Control 2. Peak enrichment in the 20 kb regions upstream of genes, coding regions, and 5′-untranslated regions (5′UTRs), was higher in the experimental DNA than in the two control samples (Figure S2C) (p value<1e-04, software MACS 1.4.0). By GO function notability enrichment analysis of peak-related genes, we found that the three samples had the same proportion of GO function genes, and the number of peak-related genes identified by the Short DHS assay was significantly higher than the numbers identified by use of the two positive controls (Figure S2D).

#### Analysis of peak-related genes or ESTs in the three datasets

This analysis revealed that the three samples had 23,506 genes or ESTs in common, and 4600, 54 and 101 unique genes or ESTs were obtained from the DNA of the Short DHS assay, control 1 and control 2, respectively (Figure S2E). The data from the Short DHS assay also contained more gene-related reads in upstream 20 K, coding, 5′UTR and 3′UTR regions than the two controls (Figure S2F). It also yielded more DHS-associated and GO-related special genes (2477 GO-related genes in biological_process) than the two controls (97 GO-related genes in biological_process in control 1 and 104 in control 2). This result indicates that Short DHS assay is a sensitive and specific method for identifying DHSs (Table S5).

### Distribution of DHSs on different chromosomes

#### Location of DHSs on different chromosomes

**Figure 4:**
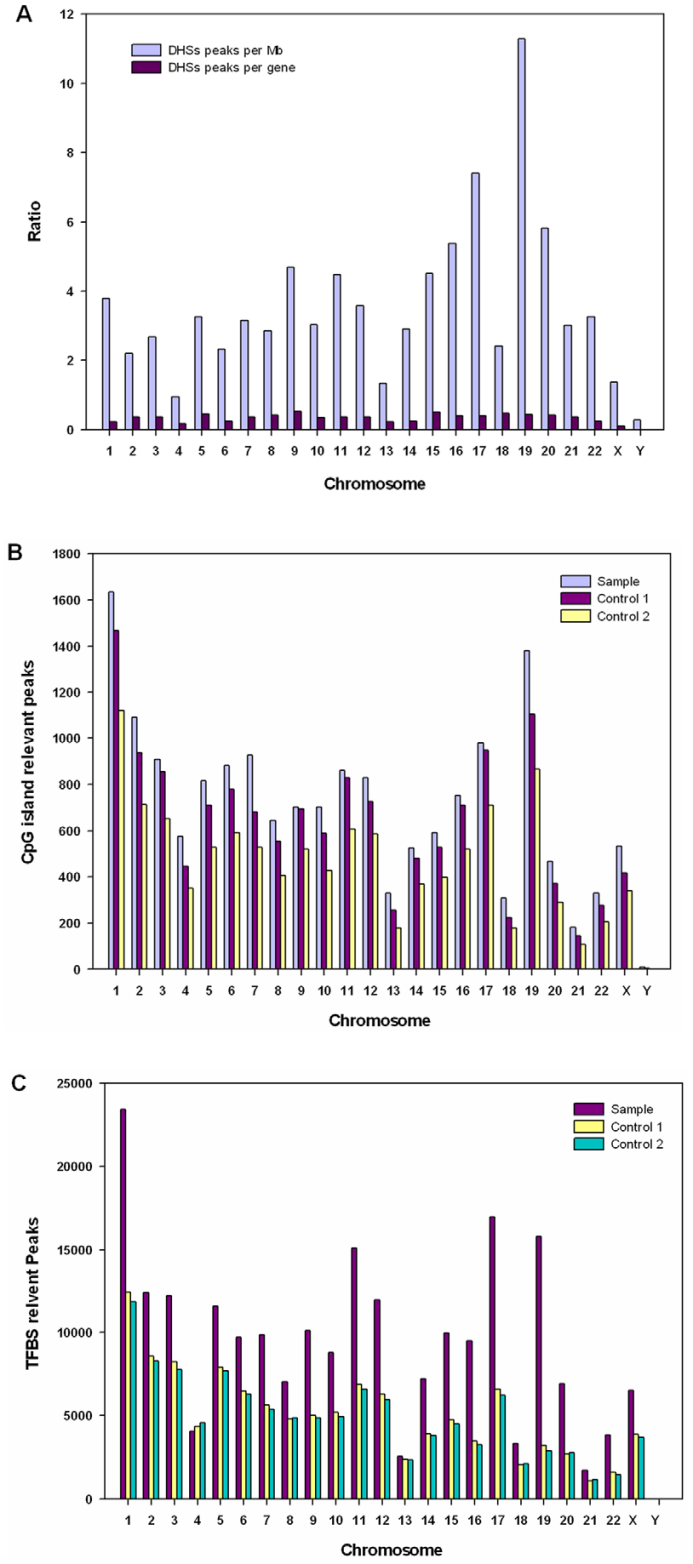
Distribution of DHSs, CpG islands, and transcription factor binding site (TFBS)-associated DHSs on different chromosomes. (A) Distribution of DHSs on the annotated genome. DHS peaks were mapped to each chromosome, and the densities of sites per Mb were determined (blue bars). (B) CpG island-related peaks on different chromosomes in the experimental sample, and controls 1 and 2. Controls 1 & 2 are two independent HeLaS3 DHS datasets downloaded from the UCSC website: DNaseI Hypersensitivity by Digital DNaseI from ENCODE/University of Washington [http://genome.ucsc.edu/cgi-bin/hgTrackUi?db=hg19&g=wgEncodeUwDnase]. Numbers of CpG island-associated peaks in the experimental sample were significantly higher than in the two positive controls. (C) TFBS-related DHS peaks on the chromosomes from the experimental Short DHS DNA (Sample), and controls 1 and 2. Numbers of TFBS-associated DHS peaks were significantly higher in the Short DHS DNA than in the two controls.

To further demonstrate that enrichment of 100–300 bp DNA fragments is an efficient and sensitive method for identifying DHSs, we mapped the locations of DHSs relative to chromosomes, CpG islands, and transcription factor binding site (TFBS). This showed that DHS peaks were significantly overrepresented on chromosomes 19 and 17, which are known to be especially gene-rich (Figure 4A blue bars). This finding is consistent with the report of Crawford et al. [10]. We found that the density of DHS peaks per gene varied between chromosomes. The number of DHS peaks per gene on chromosomes 9 and 15 reached 0.5, but was only 0.1 in the X chromosomes and chromosome 4 (Figure 4A red bars).

### *DHSs and CpG islands*

In mammalian genomes, CpG islands are in or near approximately 40% of promoters [30]. About 70% of human promoters have a high CpG content, and in vertebrates CpG islands typically occur at or near the transcription start sites of genes, particularly housekeeping genes, [31], [32]. So CpGs appear to be involved in the regulation of gene expression. Some DHS sites have been found to be associated with CpG islands. We captured more DHS-associated CpG islands using the Short DHS assay than using either of the two controls (http://genome.ucsc.edu/cgi-bin/hgTrackUi?db=hg19&g=wgEncodeUwDnase). We captured 1,379 DHS-associated CpG islands on chromosomes 19, while Control 1 captured 1,103, and control 2,867 (Figure 4B). We found that 35.7% of the DHS peaks overlapped with CpG islands, but only 13.81% of DHS peaks contained CpG islands (Table S6). We also calculated the numbers of CpG islands and CpG island-containing DHS peaks on different chromosomes. CpG island-associated DHS peaks were overrepresented on chromosome 1, 19 and 2, but under-represented on chromosomes 21, 18 and 13 (Figure 4B). These findings indicate that the DHS distribution varies between chromosomes (Figure 4B). We also found more TFBS-associated DHS peaks on chromosomes 1, 11, 17 and 19. For example, we captured 23,417 TFBS-associated DHS peaks on chromosome 1 with the Short DHS assay, while Controls 1 and 2 captured 12,426 and 11,843, respectively (Figure 4C).

#### p300 and CTCF binding sites overlap with DHSs

DNase-seq can identify all types of regulatory elements in a single experiment, however, it cannot directly reveal the function of the identified nucleosome-depleted regions, or the regulatory proteins that are bound to them. ChIP-seq could provide a degree of functional annotation. We compared Short DHS with ChIP-seq data specific to enhancer binding protein p300 and the insulator factor CTCF from the UCSC Genome Bioinformatics Site (http://genome.ucsc.edu/cgi-bin/hgTables?command=start). We investigated overlaps between p300 or CTCF binding sites and DHSs. We found that 4802 out of 29985 p300 binding sites, 19155 out of 135246 CTCF binding sites overlap with DHSs in HeLa S3 genome (Table S7 and S8), which supported the possibility that some of the DHSs we identified could be enhancers or silencers.

### DHS locations and gene expression levels on different chromosomes

**Figure 5:**
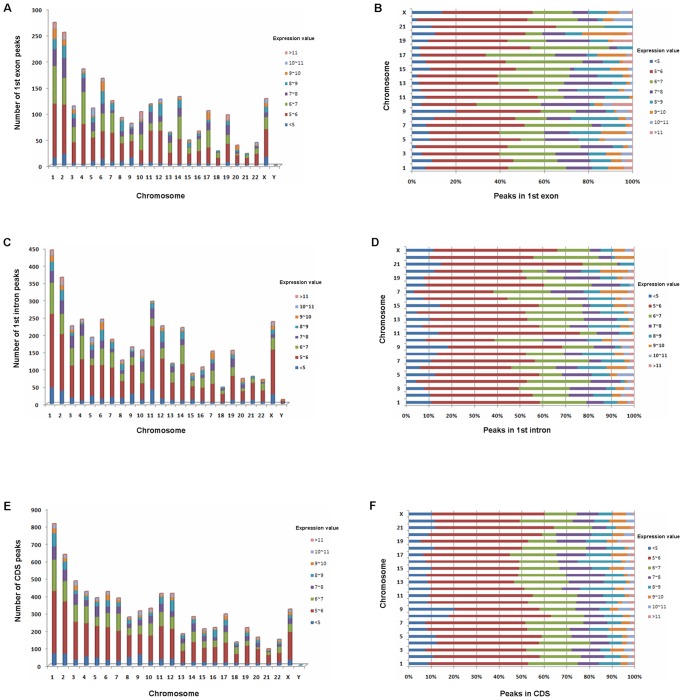
Correlations between numbers and locations of DHSs and gene expression levels on different chromosomes. (A) Numbers of first exon-associated DHS peaks. (B) Proportion of first exon-associated DHSs in genes with different expression levels on different chromosomes. (C) Overall numbers of first intron-associated DHS peaks on different chromosomes. (D) Proportion of first intron-associated DHS peaks in gene regions transcribed at different levels, on different chromosomes. (E) Numbers of coding sequence (CDS)-related DHS peaks on different chromosomes. (F) Proportion of CDS-associated DHS peaks in gene regions with different levels of transcription, on different chromosomes.

There are first exon-associated DHS peaks on all chromosomes. A relatively high proportion of first exon-associated DHSs were identified on chromosomes 1, 2, 4, 14, and X (Figure 5A). We found that 20% of the silent genes (log2<5) on chromosome 9 had DHSs in their first exon regions, but only 2% on chromosome 4) (Figure 5B). This suggests that the distribution of first exon-associated DHSs among similarly expressed genes differs on different chromosomes. We also looked at first intron and coding sequence (CDS)-related DHSs, and found marked differences in the DHS distributions at these locations on different chromosomes even for genes with similar expression levels (Figure 5C–F).

### Overall correlation between DHS distribution and gene expression

#### The distribution of DHSs in promoter region and CpG islands is positively correlated with gene expression levels

HeLa S3 DHSs were significantly enriched at promoter sites, CpG islands, downstream 20 k regions, GC boxes, and sequences from TSSs to TESs. Most of the DHS peaks were found in genes with expression values of 5–6 (Table S6). This is because genes with these expression levels constitute a large proportion of all genes and ESTs (18659 of a total of 31436 genes or ESTs; Table S6).

**Figure 6:**
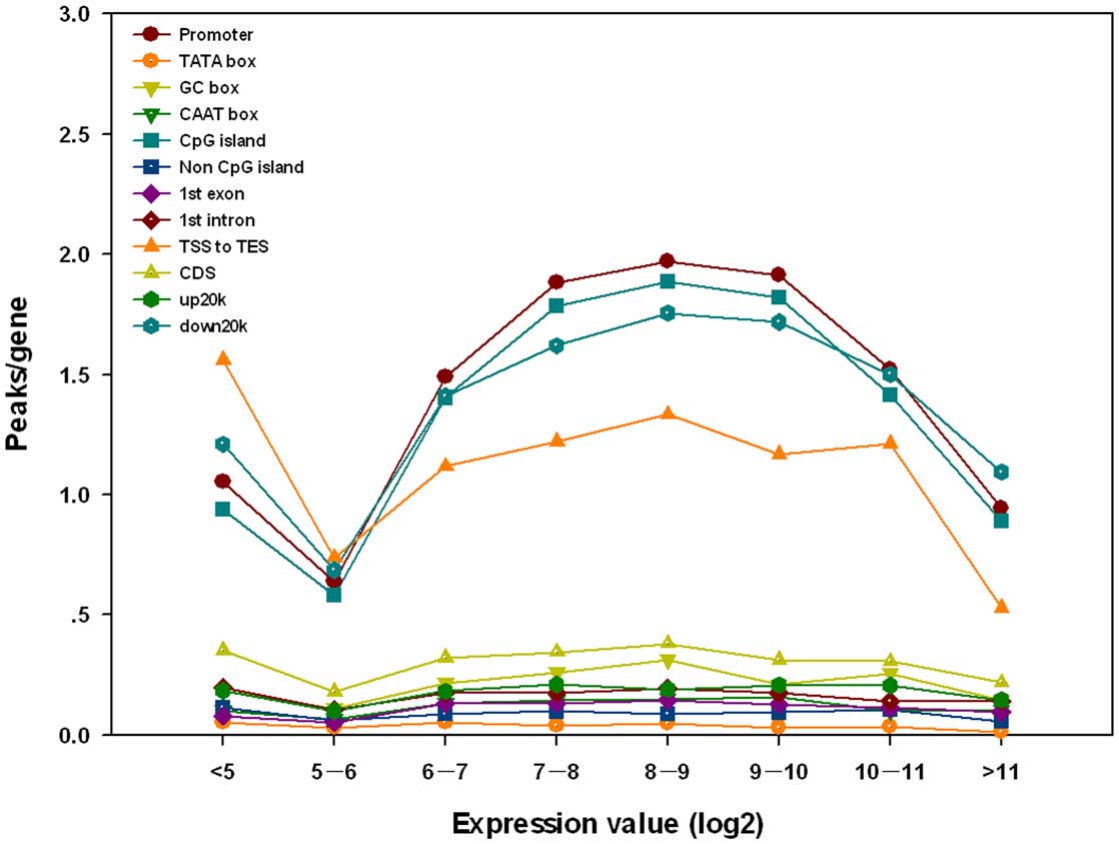
Densities of DHS peaks associated with cis-regulatory sequences/functional gene regions with different levels of expression. The logarithm base 2 values of the expression ratios were used as expression levels.

We also compared DHS-positive rates in the cis-elements and functional regions of genes with different expression levels. To our surprise we found that silenced genes (expression value <5) had a high DHS-positive rate in almost all gene-related cis-elements and functional regions (Figure 6). Since DHSs reflect the local openness and accessibility of chromatin, this indicates that the chromatin associated with silenced genes is more open than previously thought. Thus, the DHS distribution is not a simple reflection of transcription rates.

We did see some positive correlation between DHS peaks in cis-elements/functional regions of genes and level of gene expression (Figure 6). Genes with expression values of 8–9 had the highest DHS positive rate (Figure 6). In genes with very high expression levels (>10), the DHS positive rate tended to fall again (Figure 6; Table S9).

### *3′UTR DHSs are negatively correlated with active expression of genes*

**Figure 7:**
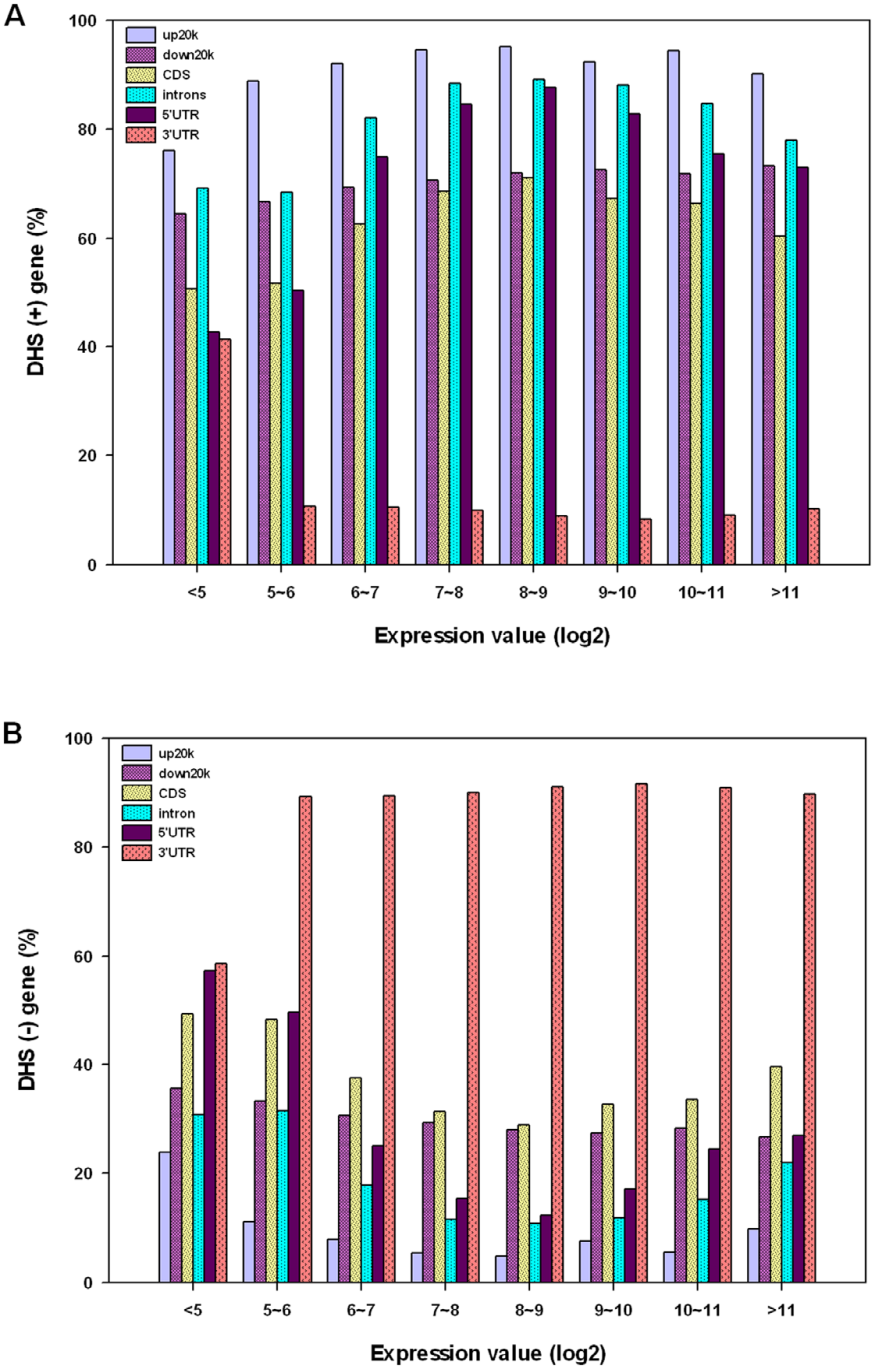
Correlations between DHS positive and negative cis-actiing elements/functional regions and gene expression levels. (A) Percentage of DHS-positive cis-acting elements/functional regions in genes with different gene expression levels. (B) Percentage of DHS-negative upstream 20 k (up20 k) regions, introns, 5′UTR, and downstream 20 k (down20 k) regions at different expression levels.

We considered the possibility that DHSs located in different cis-regulatory sequences or functional regions might play different roles in gene expression. We investigated all of the genes or ESTs that appeared to have or lack DHSs in the various cis-elements/functional regions. We found that only 10% of expressed genes (log2≥5) had DHSs in their 3′UTR regions, compared with 40% in the 3′UTRs of sile**n**ced genes (log2<5) (Figure 7A). In other words, 90% of expressed genes appear to lack DHSs in their 3′UTR regions, whereas only 60% of silenced genes lack DHSs in 3′UTRs (Figure 7B). The results indicate that 3′UTR DHSs are negatively correlate with active gene expression.

## Discussion

Transcriptional regulation is mediated by the interplay between cis-regulatory DNA elements and trans-acting transcription factors, and is perhaps the most important mechanism for controlling gene expression [33]. The components of regulatory control in the human genome include cis-acting elements that act across immense genomic distances to influence the spatial and temporal distribution of gene expression [34]. Mapping DHSs is an accurate method for identifying the locations of functional regulatory elements [35], [36], [37]. DHSs have been shown to be associated with all types of regulatory elements, including promoters, enhancers, silencers, insulators, and locus control regions. The chromatin associated with active genes may be “loosened” by electrostatic interactions between histone tails and DNA. DNase I hypersensitivity is an indication that nucleosomes are absent or that chromatin structure is loose, and is a reflection of chromatin openness and accessibility.

### The Short DHS Assay is a reliable method of identifying DHSs

DHSs result from the binding of trans-acting factors at the site of canonical nucleosomes, with consequent alteration of the local chromatin structure and increased accessibility of core functional elements and flanking regions [38], [39]. Various studies have shown that the vicinity of a DHS is nucleosome-free. Analysis at higher resolution indicates, however, that while such sites always include segments of protein-free DNA, they can also contain internal regions associated with non-histone chromosomal proteins (NHC proteins) [40].

When digested with a concentration of DNase I that cuts preferentially at DHSs, open chromatin produces a large number of DNA fragments of between 10 bp and 100 kb. In addition to the specific DNase I fragments, DNase digested DNA also contains fragments resulting from random cutting of DNA with free ends, and fragments generated by mechanical shear. Identification of the DNase I-specific fragments is the key to successful generation of a DHSs-specific library. The short DHS fragments used for generating the NGS library in the present study have some advantages. First, since only 20–75 bp of sequence is needed to uniquely map most high-throughput sequences in the genome, the long sequences produced by previous DNase I hypersensitivity assays would need additional cloning steps, and could therefore generate an experimental bias. Methods capable of generating large numbers of short DNA fragments are thus advantageous. Second, 100–300 bp DNase I double-hit fragments of active chromatin may resist mechanical shear better than longer fragments during DNase I digestion, and are likely therefore to define true DHSs. Third, short DNA fragments are easy to purify, and therefore greatly simplify experimental procedures.

We also validated the specificity of the Short DHS assay by quantitative real-time PCR. Combining whole-genome data from both Short DHS and expression microarrays, we analyzed the distribution of DHSs in different cis-elements/functional regions of genes with different expression levels. We found that DHS reads are enriched in certain cis-activating sequences/functional fragments. Our findings thus indicate that the Short DHS assay is a valuable tool for identifying open chromatin.

### The distributions of DHSs on chromosomes vary

We found that differences exist in terms of the distribution of DHSs on different chromosomes, even in similarly expressed gene regions. The chromosomes are not randomly located in the nucleus, but are instead arranged at defined positions. Three dimensionally, chromosomes occupy specific regions of the nucleus, called “chromosome territories” [41], [42]. The DHS distribution in cis-elements, such as promoters, introns, 5′ UTRs and 3′ UTRs, appears to reflect the openness of the chromatin at these sites. Even in similarly expressed gene loci we found variation between chromosomes in terms of cis-element usage and gene expression. It is possible that each chromosome territory contains a unique set of trans-activating factors or protein complexes. Further investigation of the association of different protein complexes with different cis-elements/functional regions in different chromosome territories is needed to understand the transcription network in normal cells and disease.

Chromosome territory-associated chromosome subdomains might be doing much more than just keeping everything organized. Indeed, researchers have manipulated the localization of chromosomes and seen changes in gene expression, suggesting a possible connection between chromosomal territories and disease [43]. The specific localization of chromosomes in the nucleus may indicate that they interact with different sets of trans-activation factors or epigenetic effectors. We suspect there is a connection between DHSs and chromosome territories. Thus, the different DHS distributions in different cis-activating elements/functional fragments may reflect variation in how the genes interact with trans-activation factor complexes, and differences in the protein complexes in different chromosome territories.

### Silenced genes have higher chromatin openness than low expressed genes

When we simply divided the expression of genes or ESTs into low (<6), medium (6–9) or high (9–11), we observed a positive correlation between DHSs and expression level (data not shown), in agreement with previous findings [12]. However if the genes or ESTs were classified into a larger number of subgroups, something new emerged. We found that many silent genes were associated with DHSs. This means that chromatin domains associated with silenced genes are more open and accessible than we previously thought. Thus, in contrast to previous ideas, our results support a model in which gene silencing is not associated with a stable condensed form of chromatin, but an open one.

The chromosomes of higher eukaryotes are usually subdivided into discrete functional domains in which gene expression is either repressed or facilitated. In current models repressed genes are thought to be packed in inactive chromatin, often described as condensed chromatin [44], [45]. Condensed chromatin domains are inaccessible to DNA-modifying reagents, and contain hypoacetylated histones and methylated DNA. Active or potentially active genes are packaged into a form of chromatin, referred to as euchromatin, which is more nuclease sensitive. Recently, high-resolution techniques have permitted new insights into nuclear architecture and its relationship to gene expression [46], [47]. Our results suggest that the silenced and inactive chromatin compartments are not condensed and closed, but retain a certain degree of openness, and thus may be accessible. We suspect that there is no definite constraint shielding the promoter or other functional fragments of silenced genes from external factors and that soluble nuclear protein, such as a transcription factor, can gain access to them. This dynamic situation may imply that gene silencing is not just a switch, but rather a continuous and dynamic process. This suggests that the previous view of “silenced genes” must be refined. Chromatin-associated silenced genes are actually dynamic and not “silenced”. Silenced gene loci may consist of dynamic collection of components just like active loci. The entire genome organization, irrespective of its transcriptional state, is probably in constant flux. Recently, stem cells called iPS have been produced by reprogramming genes in differentiated cells [48], [49], [50], [51]. Also, neuronal cells have been produced by trans-differentiation from fibroblasts [52], [53]. Thus, the openness of silenced gene loci would provide an appealing explanation for the plasticity of gene expression and the possibility of reprogramming.

We also found that the presence of DHSs in 3′UTRs seems to be inversely correlated with level of gene expression. Forty percent of silenced genes had associated DHSs in their 3UTR region, compared with only 10% of all expressed genes, regardless of their expression level. That is, 90% of expressed genes did not have DHSs in their 3′UTR regions. Thus, 3′UTR may act as repressors of gene expression, and DHSs in different cis-activating elements/functional DNA regions may play different or even opposite roles in gene expression.

The present study demonstrates the reliability of the Short DHS assay for identifying open chromatin. We found differences in the distribution of DHSs in different cis-elements/functional DNA regions on different chromosomes. We also found that chromatin associated with silenced gene is not condensed, as generally thought, but open and dynamic. Our results also suggest that 3′UTRs play a negative role in gene activation. We conclude that the Short DHS assay is a simple and reliable tool for DHS studies.

## Supporting Information

Figure S1 Genome-wide coverage of unique mapped reads. (A) Unique mapped reads in gene and intergenic regions. The reads in the defined regions, including the genes, gene intron, gene exon, upstream and downstream distribution of 4,000 (4 k) bps (up4 k and down4 k) were obtained using UCSC browser and converted to proportion of the total reads. (B) Proportion of unique mapped reads in different repeats is shown.
(TIF)

Figure S2 Genome-wide distribution of DNase-seq peaks. (A) Length of peaks. X-axis represents the length of peak; Y-axis represents the number of peaks. (B) Proportion of peaks with different peak hights (reads). Coverage of reads in peak regions was calculated. The read number of each peak and peak numbers were added with the cumulative statistics. That is, if a peak region contains 50 reads, in the figure all the peaks with less than or equal to 50 reads were included for the calculation of proportion of the peak with 50 reads. (C) The locations of DHSs relative to gene annotations. Genome-wide distributions of DHS peaks in annotated gene regions from three datasets are shown. DHS peaks in intergenic, intronic, downstream20 K (down20 k), upstream20 K (up20 k) and coding region were counted. (D) GO enrichment analysis of DHSs peak-related genes. The figure shows the enrichment of GO. X axis represents the GO catagories of genes; Y1 represents the proportion of GO-related genes; Y2 represents the number of GO-related genes. (E) Venn diagram shows overlap of DHSs peak-related genes or ESTs from three detasets including detaset from this study (Sample), Control 1 and Control 2. The number of total genes or ESTs and unique genes from the current study (sample group) is larger than two controls. (F) Read coverage depth in different functional regions among three datasets, including the datasets from current study and two positive controls from UCSC database. Enrichment value of DHS reads associated with upstream 20 K, CDS (coding sequence), 5′UTR and 3′UTR regions of current data obtained with Short-DHSs assay is higher than two positive control samples.
(TIF)

Table S1 Real-time PCR primer sets.
(DOC)

Table S2 Basic biological information analysis of sequencing.
(DOC)

Table S3 Genome-wide distribution of sequence reads.
(DOC)

Table S4 Genome-wide peak statistics.
(DOC)

Table S5 GO enrichment analysis of peak-relative genes for our dataset and two positive controls.
(DOC)

Table S6 Global distribution of DHS-associated with cis-regulatory elements or functional regions in the genes with different expression levels.
(DOC)

Table S7 Distribution of p300 associated DHSs over different chromosomes.
(DOC)

Table S8 Distribution of CTCF associated DHSs over different chromosomes.
(DOC)

Table S9 The DHS peak density in cis-regulatory elements or functional regions of genes with different expression value (log2).
(DOC)
