# Supplementary figures and images for: Correlation Between DNase I Hypersensitive Site Distribution and Gene Expression in HeLa S3 Cells
Source: PLoS One. 2012 Aug 10;7(8):e42414. doi: 10.1371/journal.pone.0042414 (PMC3416863; doi:10.1371/journal.pone.0042414)

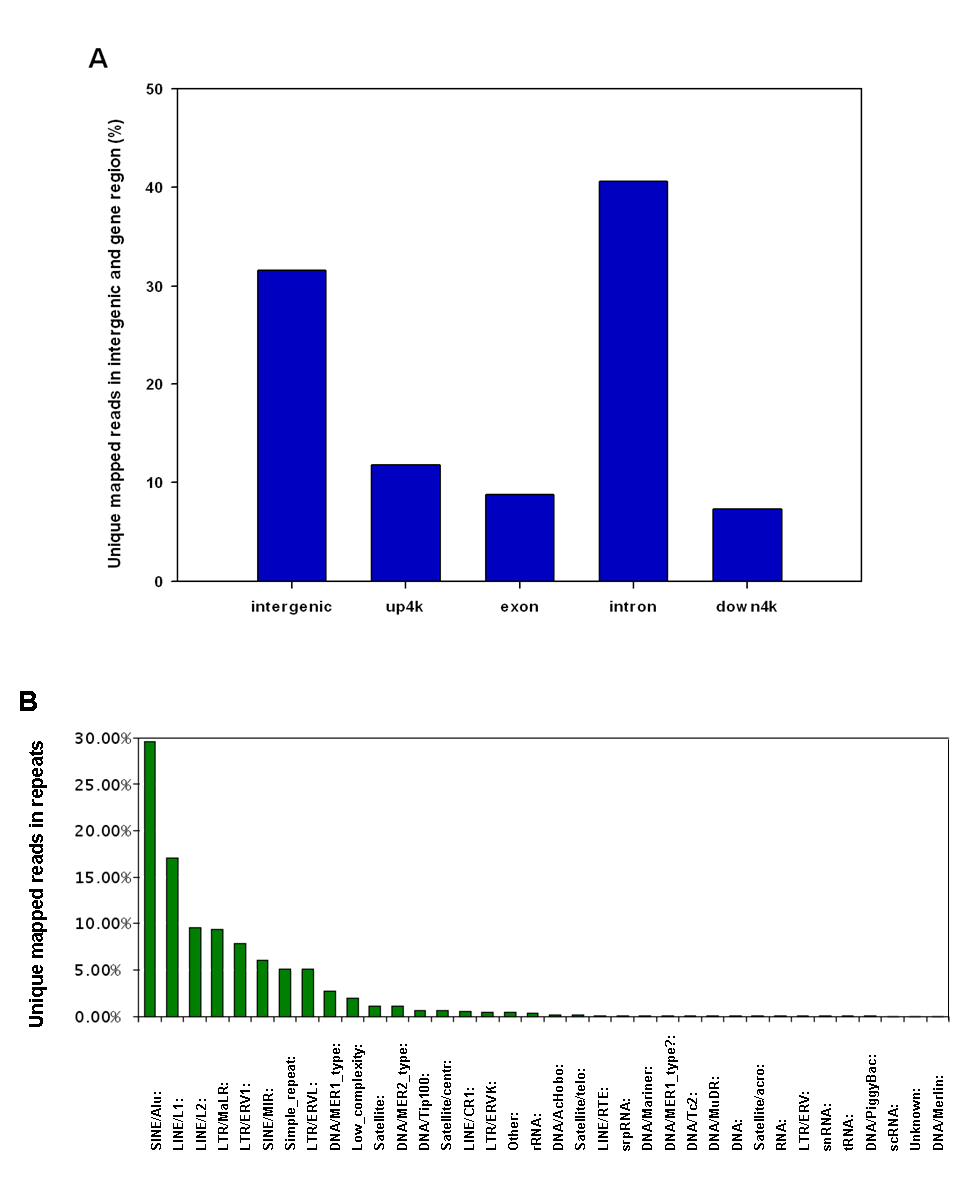

Supplement: Figure S1 — Genome-wide coverage of unique mapped reads. (A) Unique mapped reads in gene and intergenic regions. The reads in the defined regions, including the genes, gene intron, gene exon, upstream and downstream distribution of 4,000 (4 k) bps (up4 k and down4 k) were obtained using UCSC browser and converted to proportion of the total reads. (B) Proportion of unique mapped reads in different repeats is shown. (TIF) [file pone.0042414.s001.tif]

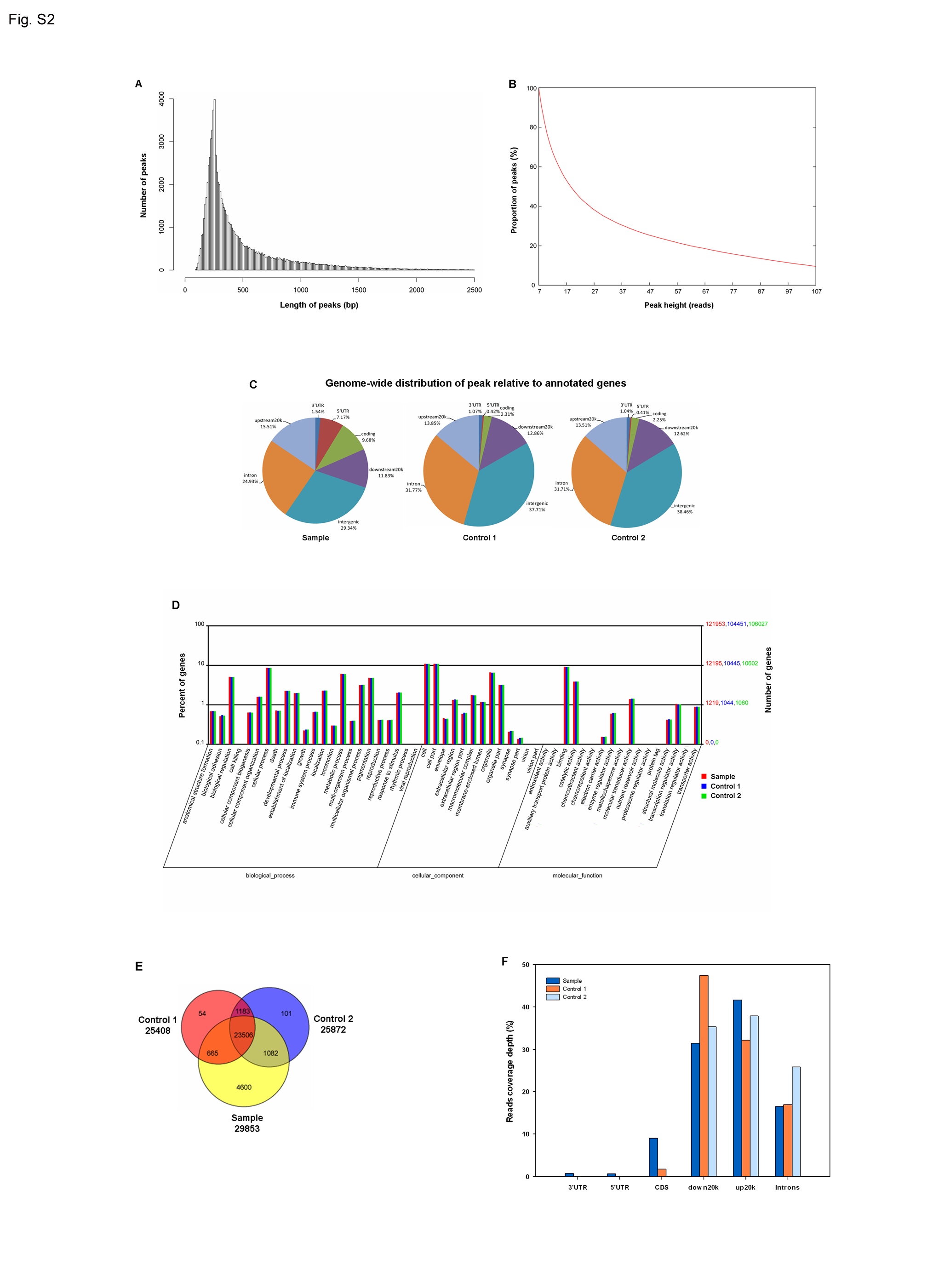

Supplement: Figure S2 — Genome-wide distribution of DNase-seq peaks. (A) Length of peaks. X-axis represents the length of peak; Y-axis represents the number of peaks. (B) Proportion of peaks with different peak hights (reads). Coverage of reads in peak regions was calculated. The read number of each peak and peak numbers were added with the cumulative statistics. That is, if a peak region contains 50 reads, in the figure all the peaks with less than or equal to 50 reads were included for the calculation of proportion of the peak with 50 reads. (C) The locations of DHSs relative to gene annotations. Genome-wide distributions of DHS peaks in annotated gene regions from three datasets are shown. DHS peaks in intergenic, intronic, downstream20 K (down20 k), upstream20 K (up20 k) and coding region were counted. (D) GO enrichment analysis of DHSs peak-related genes. The figure shows the enrichment of GO. X axis represents the GO catagories of genes; Y1 represents the proportion of GO-related genes; Y2 represents the number of GO-related genes. (E) Venn diagram shows overlap of DHSs peak-related genes or ESTs from three detasets including detaset from this study (Sample), Control 1 and Control 2. The number of total genes or ESTs and unique genes from the current study (sample group) is larger than two controls. (F) Read coverage depth in different functional regions among three datasets, including the datasets from current study and two positive controls from UCSC database. Enrichment value of DHS reads associated with upstream 20 K, CDS (coding sequence), 5′UTR and 3′UTR regions of current data obtained with Short-DHSs assay is higher than two positive control samples. (TIF) [file pone.0042414.s002.tif]
