# Supplementary material for: Correlation Between DNase I Hypersensitive Site Distribution and Gene Expression in HeLa S3 Cells
Source: PLoS One. 2012 Aug 10;7(8):e42414. doi: 10.1371/journal.pone.0042414 (PMC3416863; doi:10.1371/journal.pone.0042414)
Supplement: Table S1 — Real-time PCR primer sets. (DOC) [file pone.0042414.s003.doc]

Table S1. Real-time PCR primer sets

| Primer Name | Primer Sequence | Genome Region |
| --- | --- | --- |
| Primer set 1F | 5’-TGATGTGGCTACCAAGGTGA-3’ | chr7: 25131599-25131755 |
| Primer set 1R | 5’-GCTGGCTCCATTCAAACAAA-3’ |
| Primer set 2F | 5’-TGCAGACTTGTCCCTCACTA-3’ | chr14: 99015938-99017888 |
| Primer set 2R | 5’-GTTCCATAGCTCAGCCATAC-3’ |
| Primer set 3F | 5’-TGTAATCCCAGCACTTTCGG-3’ | chr1: 43084522-43085451 |
| Primer set 3R | 5’-TTCTCCATGTTTGTCAGGCT-3’ |
| Primer set 4F | 5’-GGGGAGAACAGGAGTTAGTCA-3’ | chr6: 36669510-36669634 |
| Primer set 4R | 5’-GGATTCCAGTCCCCATGCAC-3’ |
| Primer set 5F | 5’-AGTGCCCACAATCACAGGTT-3’ | chr11: 93866489-93867254 |
| Primer set 5R | 5’-TGCCGCAAAGACTACCAAAG-3’ |
| Primer set 6F | 5’-GGGGACTTCTTGCCAAAG-3’ | chr19: 2101777-2103067 |
| Primer set 6R | 5’-ATGTTGTCCGCCGTGTAG-3’ |

Where indicated, ‘F’ denotes forward primer and ‘R’ reverse. Primers are shown in 5’ to 3’ orientation.
