# Supplementary material for: Correlation Between DNase I Hypersensitive Site Distribution and Gene Expression in HeLa S3 Cells
Source: PLoS One. 2012 Aug 10;7(8):e42414. doi: 10.1371/journal.pone.0042414 (PMC3416863; doi:10.1371/journal.pone.0042414)
Supplement: Table S2 — Basic biological information analysis of sequencing. (DOC) [file pone.0042414.s004.doc]

Table S2. Basic biological information analysis of sequencing

| Length of read | Total reads | Production(bp) | Mapped reads | Unique mapped reads | Mapped rate | Unique mapped rate |
| --- | --- | --- | --- | --- | --- | --- |
| 35 | 14284385 | 499953475 | 13935811 | 10505670 | 97.56% | 73.55% |

Software: Eland; Reference sequence: HUMAN_refseq (hg18) [<http://hgdownload.cse.ucsc.edu/goldenPath/hg18/ chromosomes/> ]; Mapped reads means the number of which can be mapped to genome; Mapped rate = Mapped reads/total reads; Unique mapped rate = Unique mapped reads/total reads.
