# Supplementary material for: Correlation Between DNase I Hypersensitive Site Distribution and Gene Expression in HeLa S3 Cells
Source: PLoS One. 2012 Aug 10;7(8):e42414. doi: 10.1371/journal.pone.0042414 (PMC3416863; doi:10.1371/journal.pone.0042414)
Supplement: Table S3 — Genome-wide distribution of sequence reads. (DOC) [file pone.0042414.s005.doc]

Table S3. Genome-wide distribution of sequence reads

|  | | Feature | | |
| --- | --- | --- | --- | --- |
|  |  | Mappable portion of ref-genome | Exons | Introns |
| Genome-wide | | 2858032941bp | 39822496 | 992341907 |
| Proportion of mappable genome | | 100% | 1.39% | 34.72% |
| Short DHS reads | Total read base | 367698450bp | 32406185bp | 149263205bp |
|  | Proportion of read base | 100% | 8.81% | 40.59% |
|  | Enrichment^+^ | 1.0 | 6.3 | 1.2 |

Genome-wide distribution of sequence reads in coding exons and introns. Exons refers to sequences that code for [amino acids](http://en.wikipedia.org/wiki/Amino_acids). Intron refers to the DNA sequence within a gene, and the
corresponding sequence in RNA transcripts is removed by [RNA splicing](http://en.wikipedia.org/wiki/RNA_splicing). We calculate the enrichment factor using the following equation:  (number of nucleotides mapped to defined region/number of nucleotides mapped to the genome)/(number of nucleotides comprising defined region/ number of nucleotides comprising the genome).
