# Supplementary material for: Correlation Between DNase I Hypersensitive Site Distribution and Gene Expression in HeLa S3 Cells
Source: PLoS One. 2012 Aug 10;7(8):e42414. doi: 10.1371/journal.pone.0042414 (PMC3416863; doi:10.1371/journal.pone.0042414)
Supplement: Table S4 — Genome-wide peak statistics. (DOC) [file pone.0042414.s006.doc]

Table S4. Genome-wide peak statistics

| Peak counts | Total region length (bp) | Average of peak length (bp) | Median of peak length (bp) | Percentage |
| --- | --- | --- | --- | --- |
| 83897 | 40418860 | 482 | 619 | 1.35% |

The extended candidate peak area on genome was used to obtain a certain length of the modeling region. We used Poisson distribution model for the examination, according to all unique mapped reads of a region. P value was calculated in the candidate peak area. If the p-value is less than 10e-04, a region is considered a peak. Software used: MACS 1.4.0.
