# Supplementary material for: Correlation Between DNase I Hypersensitive Site Distribution and Gene Expression in HeLa S3 Cells
Source: PLoS One. 2012 Aug 10;7(8):e42414. doi: 10.1371/journal.pone.0042414 (PMC3416863; doi:10.1371/journal.pone.0042414)
Supplement: Table S5 — GO enrichment analysis of peak-relative genes for our dataset and two positive controls. (DOC) [file pone.0042414.s007.doc]

**Table S5. GO enrichment analysis of peak-relative genes for our dataset and two positive controls**

|  | | Short DHS | Control 1 | Control 2 |
| --- | --- | --- | --- | --- |
| Unique part | Biological_process | 2477 | 97 | 104 |
|  | Cellular_component | 370 | 44 | 44 |
|  | Molecular_function | 701 | 29 | 53 |
| Common part | Biological_process | 4854 | | |
|  | Cellular_component | 690 | | |
|  | Molecular_function | 1789 | | |
