# Supplementary material for: Correlation Between DNase I Hypersensitive Site Distribution and Gene Expression in HeLa S3 Cells
Source: PLoS One. 2012 Aug 10;7(8):e42414. doi: 10.1371/journal.pone.0042414 (PMC3416863; doi:10.1371/journal.pone.0042414)
Supplement: Table S6 — Global distribution of DHS-associated with cis-regulatory elements or functional regions in the genes with different expression levels. (DOC) [file pone.0042414.s008.doc]

**Table S6. Global distribution of DHS-associated with cis-regulatory elements or functional regions in the genes with different expression levels**

| Expression value (log2) | | <5 | 5-6 | 6-7 | 7-8 | 8-9 | 9-10 | 10-11 | >11 | Total |
| --- | --- | --- | --- | --- | --- | --- | --- | --- | --- | --- |
| Gene number | Total expression genes (or ESTs) | 2109 | 18659 | 4498 | 2438 | 1638 | 970 | 534 | 580 | 31426 |
| Peak number | Promoter | 2219 | 11911 | 6695 | 4585 | 3227 | 1856 | 811 | 546 | 32019 |
|  | TATA box | 104 | 556 | 221 | 92 | 77 | 29 | 18 | 6 | 1582 |
|  | GC box | 391 | 2015 | 963 | 627 | 508 | 202 | 136 | 82 | 6211 |
|  | CAAT box | 213 | 1174 | 585 | 355 | 244 | 150 | 52 | 59 | 3666 |
|  | CpG island | 1979 | 10811 | 6313 | 4349 | 3086 | 1766 | 756 | 515 | 29719 |
|  | Non CpG island | 240 | 1100 | 382 | 236 | 141 | 90 | 55 | 31 | 2300 |
|  | Gene regions from TSS to TES | 3291 | 13744 | 5019 | 2972 | 2182 | 1132 | 647 | 306 | 29293 |
|  | 1st exon | 167 | 978 | 582 | 322 | 238 | 124 | 60 | 55 | 3313 |
|  | 1st intron | 414 | 1957 | 782 | 427 | 317 | 169 | 75 | 81 | 6819 |
|  | CDS(coding sequence) | 741 | 3319 | 1441 | 837 | 621 | 301 | 163 | 126 | 9340 |
|  | Upstream 20k | 386 | 1839 | 816 | 510 | 310 | 201 | 109 | 84 | 6204 |
|  | Downstream 20k | 2551 | 12788 | 6343 | 3950 | 2875 | 1667 | 801 | 634 | 41459 |
