# Supplementary material for: Correlation Between DNase I Hypersensitive Site Distribution and Gene Expression in HeLa S3 Cells
Source: PLoS One. 2012 Aug 10;7(8):e42414. doi: 10.1371/journal.pone.0042414 (PMC3416863; doi:10.1371/journal.pone.0042414)
Supplement: Table S7 — Distribution of p300 associated DHSs over different chromosomes. (DOC) [file pone.0042414.s009.doc]

**Table S7. Distribution of p300 associated DHSs over different chromosomes**

| Chromosome | Number of p300 bindng sites | Control 1 | Control 2 | Short DHS |
| --- | --- | --- | --- | --- |
| chr1 | 2790 | 107 | 114 | 518 |
| chr2 | 2508 | 79 | 87 | 350 |
| chr3 | 2162 | 71 | 77 | 312 |
| chr4 | 1537 | 39 | 39 | 101 |
| chr5 | 2147 | 105 | 113 | 299 |
| chr6 | 1977 | 84 | 89 | 261 |
| chr7 | 1596 | 64 | 68 | 255 |
| chr8 | 1444 | 68 | 64 | 228 |
| chr9 | 1379 | 56 | 57 | 235 |
| chr10 | 1449 | 82 | 81 | 220 |
| chr11 | 1562 | 75 | 77 | 272 |
| chr12 | 1499 | 52 | 51 | 214 |
| chr13 | 812 | 15 | 22 | 64 |
| chr14 | 863 | 29 | 25 | 121 |
| chr15 | 1000 | 29 | 28 | 221 |
| chr16 | 690 | 23 | 31 | 193 |
| chr17 | 942 | 42 | 47 | 221 |
| chr18 | 789 | 26 | 29 | 113 |
| chr19 | 445 | 19 | 21 | 160 |
| chr20 | 723 | 36 | 32 | 177 |
| chr21 | 425 | 10 | 15 | 80 |
| chr22 | 328 | 10 | 10 | 75 |
| chrX | 918 | 30 | 35 | 112 |
| Total | 29985 | 1151 | 1212 | 4802 |
