# Supplementary material for: Correlation Between DNase I Hypersensitive Site Distribution and Gene Expression in HeLa S3 Cells
Source: PLoS One. 2012 Aug 10;7(8):e42414. doi: 10.1371/journal.pone.0042414 (PMC3416863; doi:10.1371/journal.pone.0042414)
Supplement: Table S8 — Distribution of CTCF associated DHSs over different chromosomes. (DOC) [file pone.0042414.s010.doc]

**Table S8. Distribution of CTCF associated DHSs over different chromosomes**

| Chromosome | Number of CTCF binding sites | Control 1 | Control 2 | Short DHS |
| --- | --- | --- | --- | --- |
| chr1 | 11238 | 256 | 252 | 1633 |
| chr2 | 9675 | 146 | 157 | 1143 |
| chr3 | 11190 | 199 | 222 | 1129 |
| chr4 | 3662 | 32 | 35 | 358 |
| chr5 | 9906 | 193 | 197 | 1025 |
| chr6 | 7926 | 130 | 159 | 935 |
| chr7 | 6537 | 103 | 114 | 890 |
| chr8 | 7818 | 114 | 134 | 860 |
| chr9 | 5869 | 129 | 128 | 999 |
| chr10 | 5449 | 90 | 109 | 827 |
| chr11 | 7167 | 184 | 200 | 1190 |
| chr12 | 7952 | 154 | 164 | 1129 |
| chr13 | 3047 | 29 | 29 | 271 |
| chr14 | 4667 | 70 | 65 | 633 |
| chr15 | 5169 | 101 | 109 | 851 |
| chr16 | 4530 | 83 | 85 | 866 |
| chr17 | 4685 | 122 | 126 | 1136 |
| chr18 | 3621 | 47 | 38 | 347 |
| chr19 | 3912 | 133 | 139 | 1253 |
| chr20 | 3623 | 73 | 73 | 658 |
| chr21 | 1803 | 38 | 39 | 243 |
| chr22 | 1485 | 31 | 32 | 348 |
| chrX | 4315 | 49 | 54 | 431 |
| Total | 135246 | 2506 | 2660 | 19155 |
