# Supplementary material for: Correlation Between DNase I Hypersensitive Site Distribution and Gene Expression in HeLa S3 Cells
Source: PLoS One. 2012 Aug 10;7(8):e42414. doi: 10.1371/journal.pone.0042414 (PMC3416863; doi:10.1371/journal.pone.0042414)
Supplement: Table S9 — The DHS peak density in cis-regulatory elements or functional regions of genes with different expression value (log2). (DOC) [file pone.0042414.s011.doc]

**Table S9. The DHS peak density in cis-regulatory elements or functional regions of genes with different expression value (log2)**

|  | Peak/gene | | | | | | | |
| --- | --- | --- | --- | --- | --- | --- | --- | --- |
| Expression value (log2) | <5 | 5－6 | 6－7 | 7－8 | 8－9 | 9－10 | 10－11 | >11 |
| Promoter | 1.0522 | 0.6384 | 1.4884 | 1.8806 | 1.9701 | 1.9134 | 1.5187 | 0.9414 |
| TATA box | 0.0493 | 0.0298 | 0.0491 | 0.0377 | 0.0470 | 0.0299 | 0.0337 | 0.0103 |
| GC box | 0.1854 | 0.1080 | 0.2141 | 0.2572 | 0.3101 | 0.2082 | 0.2547 | 0.1414 |
| CAAT box | 0.1010 | 0.0629 | 0.1301 | 0.1456 | 0.1490 | 0.1546 | 0.0974 | 0.1017 |
| CpG island | 0.9384 | 0.5794 | 1.4035 | 1.7838 | 1.8840 | 1.8206 | 1.4157 | 0.8879 |
| Non-CpG island | 0.1138 | 0.0590 | 0.0849 | 0.0968 | 0.0861 | 0.0928 | 0.1030 | 0.0534 |
| 1st exon | 0.0792 | 0.0524 | 0.1294 | 0.1321 | 0.1453 | 0.1278 | 0.1124 | 0.0948 |
| 1st intron | 0.1963 | 0.1049 | 0.1739 | 0.1751 | 0.1935 | 0.1742 | 0.1404 | 0.1397 |
| TSS to TES | 1.5605 | 0.7366 | 1.1158 | 1.2190 | 1.3321 | 1.1670 | 1.2116 | 0.5276 |
| CDS (coding sequence) | 0.3514 | 0.1779 | 0.3204 | 0.3433 | 0.3791 | 0.3103 | 0.3052 | 0.2172 |
| Up20k | 0.1830 | 0.0986 | 0.1814 | 0.2092 | 0.1893 | 0.2072 | 0.2041 | 0.1448 |
| Down20k | 1.2096 | 0.6854 | 1.4102 | 1.6202 | 1.7552 | 1.7186 | 1.5000 | 1.0931 |
